# Key Teacher Attitudes for Sustainable Development of Student Employability by Social Cognitive Career Theory: The Mediating Roles of Self-Efficacy and Problem-Based Learning

**DOI:** 10.3389/fpsyg.2020.01945

**Published:** 2020-09-30

**Authors:** Xiang Liu, Michael Yao-Ping Peng, Muhammad Khalid Anser, Wei-Loong Chong, Biqu Lin

**Affiliations:** ^1^School of Economics and Management, Fuzhou University of International Studies and Trade, Fuzhou, China; ^2^School of Economics and Management, Foshan University, Foshan, China; ^3^School of Digital Economics, Guilin University of Electronic Technology, Guilin, China; ^4^School of Public Administration, Xi’an University of Architecture and Technology, Xi’an, China; ^5^Department of Education, New Era University College, Kajang, Malaysia; ^6^School of Marxism, Fujian Normal University, Fuzhou, China

**Keywords:** self-efficacy, social cognitive career theory, problem-based learning, employability, transformational leadership

## Abstract

Higher education policy and talent training are failing to meet the ever-changing expectations of employers and society in Taiwan, resulting in a gap between university education and employment. This study used social cognitive career theory (SCCT) to explore the relationships among self-efficacy, transformational leadership (TL), student employability (SE), and problem-based learning (PBL) in higher education institutions (HEIs). The analysis of 637 undergraduates from 16 Taiwanese HEIs using structural equation modeling (SEM) shows significant positive correlations among self-efficacy, PBL, TL, and SE, with PBL and self-efficacy as key mediators. Based on these findings, the researchers propose feasible suggestions for related issues and future research.

## Introduction

Scholars in multiple disciplines have focused on the concept of student employability (SE). Studies have confirmed the importance of the development of SE. De Vos et al. (2011) suggested that SE is acquired by students through developing skills, knowledge, and capacity to meet the talent demands from the employment market. Various research frameworks have provided valuable insights and contributions concerning SE issues. Crossman and Clarke (2010) emphasized the link between higher education and SE, stating that the strength of this relationship affects employers’ and graduates’ satisfaction with university education. However, most studies have discussed employee performance in the workplace (Cuyper et al., 2008), neglecting the fact that the cultivation of SE depends on the curriculum design provided by lecturers. Indeed, universities must improve SE through factors or modes of operation that remain to be clarified, and students’ employment performance can increase universities’ visibility, which is a focus of concern and investment for these institutions.

Several previous studies have suggested that skills development, knowledge acquisition, and capacity construction of university students are mostly related to the “teacher factor” (Bolkan and Goodboy, 2011; Shatzer et al., 2013; Pounder, 2014; Peng et al., 2018). That is, students need teachers to provide courses and curriculum design based on employment trends, ethical values, career planning, work features, and even SE to reduce the academia–employment gap. As positive inspiration and guidance from lecturers can facilitate the development of SE and influence students’ learning motivation, investment, and effectiveness (Robinson et al., 2008), teachers should consider undertaking leadership courses. Higher education teachers’ leadership in a class implies not only applying concepts that have been found to be beneficial to learning but also maintaining complex relationships with students, including adhering to moral responsibilities and managing emotional experiences (Kelchtermans, 2009; Scager et al., 2017). Further, promoting students’ active learning attitudes and cultivating good employment conditions also hinge on teachers’ leadership. Previous studies have considered teachers’ transformational leadership (TL; Robinson et al., 2008; Pounder, 2014). This leadership style heightens the consciousness of collective interest among students and helps them to achieve their learning goals (Pounder, 2008, 2014; Robinson et al., 2008; Bolkan and Goodboy, 2011; Shatzer et al., 2013).

According to Bandura (1997) and social cognitive theory (SCT), personal attributes, environmental influences, and intentional behaviors form a triangular relationship of interaction, wherein individual behavior is formed *via* the interaction of inner thoughts, emotions, and the environment (Peng et al., 2018). Based on SCT, social cognitive career theory (SCCT) was proposed to explain the influencing factors and development of satisfaction in education (Lent et al., 1994; Lent and Brown, 2006). In the SCCT model, there is an indirect effect of environmental factors and behavioral factors on personal cognitive factors. Thus, when personal cognitive factors are expected to directly affect SE, TL’s impact on the development of SE will be less significant. Self-efficacy is students’ belief in their successful performance and education-related behaviors and abilities, and it is an important factor in initiating spontaneous learning motivation and engagement (Parker et al., 2006), as well as being central to SCCT. Therefore, this study suggests that combining the TL, self-efficacy, and SE concepts of SCCT will help to address the deficiencies of prior literature.

Most of the factors affecting student achievement have indirect effects (Pascarella et al., 2005). In addition to strengthening student self-efficacy (SSE), teachers can use TL to design the learning context such that it promotes the development of students’ knowledge, abilities, and skills, such as problem-solving (PS), interpersonal, and communication skills. In SCCT, teachers’ leadership plays a significant role in creating a learning context (situations and outcomes) that enables students to achieve superior SE (Lent and Brown, 2006), the core of which is solving workplace-based problems. Therefore, problem-based learning (PBL) enables students to be involved in their learning environments, acquire strategic knowledge, enhance their PS capabilities, and develop their learning effectiveness (Chang et al., 2012; Peng et al., 2018), which form the foundation for developing their employability.

## Literature Review and Hypotheses Development

### Social Cognitive Career Theory

According to SCT (Bandura, 1997), individual behavior is based on the continuous interaction of individual attributes, environmental influences, and purposive behavior. That is, an occurrence of or change in behavior is rooted in the individual–environment interaction; thus, the process by which behaviors form under the influence of external and internal factors can be explained. Social cognitive career theory (Lent et al., 1994) considers the influence of students’ attributes and their background factors on behavior choice, adding self-efficacy, outcome expectation, and target selection to the model. Social cognitive career theory thus refers to the professional context. In addition to adding career choice, development of interests, and performance to the original model, SCCT emphasizes the importance of self-efficacy and learning experience for the development of employability.

Lent and Brown (2006) extended SCT and general well-being model of Lent (2004), integrating “top-down” (aptitude) and “bottom-up” (context) aspects into the study of well-being to explain the adaptation processes of individuals affected by different factors in education and occupation. They proposed three main causal paths and findings: (1) there are positive and negative effects of partial cross-context experience factors, which may directly affect satisfaction in the workplace; (2) situational and emotional factors partially mediate the path between personal traits and satisfaction; and (3) personal traits indirectly affect work/education satisfaction through self-efficacy and environmental support. Similarly, related theories have also been proposed by Sheu et al. (2014). Thus, this study explored, from the perspective of teachers, the supportive context for the class or course created by teachers *via* TL so as to verify its relationship with SE. More specifically, PBL is an important component in the learning process (Chang et al., 2012). This study attempted to verify the direct effects of PBL on SE and SSE and analyze its indirect effect on the relationship between TL and SE in the model.

### Student Employability

Student employability has attracted increasing academic attention in recent years. Van der Heijde and Van der Heijden (2006) argue that employability is an individual’s appropriate application of competence, their continuous acquisition and creation of essential work skills in order to accomplish tasks, and their ability to adapt to internal and external labor market changes (Fugate et al., 2004; Cuyper et al., 2008; Vermeulen et al., 2018; Shahzad et al., 2019). Hence, the need for critical and reflective thinking skills, problem-solving abilities, self-management skills, learning capacity, and related competencies is continually increasing across all disciplines (Makkonen, 2017). Several prior studies have indicated that in addition to the influence of basic education on employability, factors like personal attributes, interpersonal relationships, and external factors that cannot be acquired in higher education should also be considered (Ahmed et al., 2015; Cacciolatti et al., 2017; Blázquez et al., 2018).

Thus, SE can be referred to as a higher-order construct (Pan and Lee, 2011). Lees’ review of the SE skills literature (Lees, 2002) and the SE agenda (Hennemann and Liefner, 2010) revealed that personal qualities, core skills, and process skills are key for employers. Pan and Lee (2011) surveyed the flow of higher education institution (HEI) graduates in Taiwan, adopting the employability scale developed by Andrews and Higson (2008). They suggested that employability should cover the general and professional abilities required at work, work attitude, career planning ability, and confidence. The present study adopted the employability classification of Pan and Lee (2011) as the measurement of SE.

### Transformational Leadership

The concept of TL was proposed by Burns (1978), who stated that when leaders possess qualities such as mutual cooperation, enthusiasm, empowerment, vision, and creativity, they can inspire followers to achieve high motivation and strong performance and to develop shared values (Bolkan and Goodboy, 2011; Peng et al., 2018). Thus, TL is the process by which leaders communicate their emotions, attitudes, values, and beliefs to motivate subordinates (Öqvist and Malmström, 2016). Most scholars in this field have adopted the measurement variables summarized by Bass and Steidlmeier (1999) to measure the degree of TL. Specifically, (1) idealized influence (II) means that the leader can clearly express their ideas and visions to followers and encourage them to devote themselves to and participate in the realization of these visions, so there is a high degree of trust and a sense of shared identity between the followers and the leader (Harrison, 2011); (2) intellectual stimulation (IS) is a way for the leader to encourage their followers to question existing problems and is an important element in organizational learning and organizational change that also challenges the leader’s existing norms and decision-making models to improve the use of various methods (Avolio et al., 1999; Shatzer et al., 2013); (3) individualized consideration (IC) is the extent to which the leader satisfies each follower’s need for mentoring, including support, encouragement, and guidance. After considering the different attributes and traits of followers, the leader sets reasonable goals and then gives followers opportunities for growth and development through a journey of self-realization; and (4) inspirational motivation (IM) refers to the leader’s abilities regarding expression, attraction, and inspiration. It enables followers to achieve challenging and meaningful goals through the transmission and communication of ideas (Bass and Steidlmeier, 1999; Bolkan and Goodboy, 2011).

When lecturers apply TL to enhance students’ positive behaviors and attitudes, they must adopt clarified transformations of cognitive variables to demonstrate their influence (Peng et al., 2018). Therefore, this study referred to TL as an important antecedent in the SCCT model to explore the impact on the development of SE. Poekert (2012) claimed that teachers’ leadership is centered on influence and interaction, rather than power and authority. Thus, teachers create a vision for students to follow in class, causing students to remain open-minded and respectful of others, thereby improving their learning practice (Shatzer et al., 2013; Pounder, 2014). Öqvist and Malmström (2016) proposed that students’ educational motivation and performance are highly dependent on teachers’ leadership and that teachers have a position of extreme power over students, for example, regarding guidance, modeling, enthusiasm, self-efficacy, sincere praise, reinforcement, and interest induction. Teachers’ conveyance of educational concepts and learning values can greatly improve students’ commitment to learning and self-efficacy (Harrison, 2011; Pounder, 2014; Peng et al., 2018). Thus, Hypothesis 1 was proposed:

Hypothesis 1: Teachers’ TL has a positive and significant impact on students’ self-efficacy.

Student employability is more diversified than professional competence. Apart from social soft power and hard power, it also includes psychological attitudes and cognitions related to job searches. Besides, teachers must utilize intrinsic and extrinsic incentives to guide students to foster their employment skills (Harrison, 2011; Bogler et al., 2013). General and professional abilities represent students’ learning outcomes and academic performance. Thus, students must have high learning satisfaction (Bogler et al., 2013) as a basis for the development of SE. Scholars have confirmed that teachers with TL are better able to motivate students to set goals and achieve learning satisfaction (McGrath et al., 2006). In order to create learning satisfaction, teachers must offer participation opportunities to students, enhance students’ trust in them, and be willing to improve their practices (Harrison, 2011; Bogler et al., 2013; Pounder, 2014; Peng et al., 2018). Teachers use intellectual stimulation to encourage students to study in-depth, as well as to enable students to develop team awareness and to try to overcome learning difficulties under the influence of individualized consideration. Teachers’ inspirational motivation guides students to realize their potential to gain more knowledge and skills that contribute to employability. Thus, Hypothesis 2 was as follows:

Hypothesis 2: Teachers’ TL has a positive and significant impact on students’ employability.

Universities and industries have a close relationship of cultivation and cooperation. Students must gain employment knowledge before entering the workforce. Previous studies have indicated that problem-solving is one of the most important capabilities gained from learning (Van der Heijde and Van der Heijden, 2006; Cuyper et al., 2008; Pan and Lee, 2011). Problem-based learning is an effective learning model (Dunlap, 2005); however, it must be created not by students’ learning behaviors but through teachers’ guidance and curriculum design (Tagg, 2003). Bolkan and Goodboy (2011) indicated that teachers who promote participation in specific learning contexts generally facilitate individualized consideration and intellectual stimulation. By sharing opinions and collaborating with each other, students are exposed to a multitude of ideas and can address problems in new ways. By facilitating the discussion of problems, teachers can solicit feedback from individuals (individualized consideration) and promote intellectually stimulating conversations between students (Bolkan and Goodboy, 2011). Therefore, through TL, teachers can inspire students to apply different problem-solving methods to provide more-valuable insights and programs in the classroom (Pounder, 2008, 2014; Chang et al., 2012). Consequently, teachers must consider students’ learning demands and capacities in order to dynamically adjust curriculum content to transform students’ knowledge and experience, enabling them to solve problems (Robinson et al., 2008) and encouraging them to share information with each other to increase PBL (Peng et al., 2018). Thus, this study proposed Hypothesis 3 as follows:

Hypothesis 3: Teachers’ TL has a positive and significant impact on student PBL.

### Student Self-Efficacy

The concept of self-efficacy was proposed by Bandura (1997) and has been verified in different fields, especially regarding impacts on learning performance, such as the academic achievement of students (Choi, 2005; Dunlap, 2005). Nevertheless, conclusions have differed regarding the relationship between self-efficacy and outcomes. For example, Lent and Brown (2006) stated that self-efficacy positively impacts academic satisfaction, while Lent et al. (2012) found that there was no significant effect. This difference may be due to the measurement of self-efficacy. Previous studies have shown a high degree of predictive validity when task-specific self-efficacy is measured; thus, research on self-efficacy should explore its significant effects on measurable performance indicators and variables (Bandura, 1997; Choi, 2005; Peng et al., 2018). However, context-specific self-efficacy has been transformed into exclusive self-efficacy for multiple fields through repeated successes and failures in different contexts, and context-specific self-efficacy has been generalized to different tasks based on the experience of operational tasks, such as academic self-efficacy (Lent et al., 1997).

Zhao et al. (2005) used SCT and sampled 1,043 Master of Business Administration students at five universities in order to understand the relationships among personal characteristics, self-efficacy, cognitive experience, and entrepreneurial intention. Their research findings showed that students with high self-efficacy could enhance their own self-confidence and understanding, which they needed to get a job offer or start up a business. They consequently had positive work attitudes and good career planning skills. Dacre Pool and Qualter (2013) indicated that students’ initiative in the formation and application of SE is low. This can be attributed to students’ lack of motivation to acquire more employment knowledge or a lack of self-efficacy. Therefore, some scholars have suggested that students with more self-efficacy can improve their development of social connections so that they can effectively manage the interpersonal relationships needed in their future workplaces (Peng et al., 2018). Thus, Hypothesis 4 was as follows:

Hypothesis 4: Self-efficacy has a positive and significant impact on students’ employability.

According to the above arguments, teachers’ TL provides support and guidance in students’ learning. Teachers’ TL can thus effectively improve SE (Pounder, 2008, 2014; Harrison, 2011; Bogler et al., 2013). In the SCCT model, cognitive variables can enhance the supporting effect derived from TL and further the development of SE. Garriott et al. (2013) used SCCT to examine the learning interests and learning goals of first-generation university students in low-income households. They showed that self-efficacy has a significant indirect effect on support and goals, which are important mediating cognitive variables. Similarly, teachers provide the supporting elements of TL during classes, which guides students to have the motivation and confidence to develop skills and acquire related knowledge (Wang and Fu, 2015), as well as strengthens their cognition regarding career planning, thereby cultivating employability. Thus, Hypothesis 5 was proposed:

Hypothesis 5: Self-efficacy plays a mediating role between TL and SE.

### Problem-Based Learning

Problem-based learning is a learning mode that has received much attention in recent decades (Dunlap, 2005; McGrath et al., 2006; Chang et al., 2012). It emphasizes that student-oriented teaching divides the learning process into five stages: propose problem, establish hypothesis, collect data, demonstrate hypothesis, and summarize (Peng et al., 2018). In complex but meaningful problem situations, students acquire and develop the knowledge required to solve a problem through learning, and they develop the ability to learn independently (McGrath et al., 2006). Regarding the measurement of PBL, Chang et al. (2012) proposed “problem-solving” and “knowledge-sharing.” Problem-solving entails addressing problems and challenging situations by using resources to break existing thought models and recombining ideas and problem-solving plans (Peng et al., 2018). Knowledge-sharing (KS) is the establishment of a common understanding and focuses on the problem-solving process. Through the exploration and combination of ideas, knowledge is integrated and constructed to achieve shared knowledge among individuals (Peng et al., 2018).

Studies have shown that learning opportunities can positively enhance individuals’ capabilities and outcomes, thus improving their self-efficacy (Zhao et al., 2005). In order to enhance their self-efficacy, students must engage in learning experiences over a long period, which will affect their subjective assessment of actions (challenges) and individual abilities (skills) regarding environmental opportunities. That is, students’ participation in learning challenges and the development of knowledge will enhance the resources they can dedicate to learning challenges, so appropriate learning experience can be obtained. Therefore, in addition to internal incentives, the design of learning activities should encourage students to find the meaning of learning during knowledge exploration, shaping their long-term learning goals and personal career orientation (Delle Fave and Massimini, 2005).

In the learning process, greater perceived self-efficacy positively affects students’ learning motivation, cognitive capabilities, academic interests, emotion management, and achievement (Bandura, 1997; Peng et al., 2018). Self-efficacy plays a strong mediating role in the relationship between performance and self-realization (Lent and Brown, 2006). Dunlap (2005) found that PBL can enable students to effectively obtain professional knowledge and skills; however, while such knowledge can enhance learning effectiveness, if there is no self-efficacy as a prerequisite, the effect may be limited (Peng et al., 2018). Therefore, a problem-oriented learning strategy should emphasize the setting of short-term and long-term goals and give feedback on students’ learning achievements as a source of learning improvement, thereby enhancing their self-efficacy. Thus, Hypothesis 6 was proposed:

Hypothesis 6: Problem-based learning has a positive and significant impact on SSE.

Problem-based learning is helpful in enhancing students’ interests in learning and career paths. It is connected with SE, as it can facilitate students in developing the appropriate learning attitudes and higher-order thinking skills needed to face real-world challenges, such as critical thinking, problem-solving, and reflection skills (White et al., 2004). Some scholars have confirmed that students who engage in PBL strengthen their learning motivation, attitudes, and behaviors, thus improving their learning autonomy, critical thinking skills, and employability (Peng et al., 2018). Therefore, Hypothesis 7 was posited:

Hypothesis 7: Problem-based learning has a positive and significant impact on students’ employability.

In addition to being considered an important independent variable of employability, PBL may play an important mediating role in the relationship between SE and TL. Studies have found that the learning context can have a decisive influence on SE, depending on the attributes (Martin et al., 2008), so it can be seen as an important reference for learning processes and outcomes. Although teachers’ TL can enhance students’ motivation to improve their own abilities, studies have indicated that students can only build their own knowledge and develop meaningful social cognitive connections among prior knowledge, experience, and newly acquired knowledge by cooperating with other learners and teachers on certain tasks (Mierson and Freiert, 2004), thereby improving their employability. In other words, PBL may have a mediating effect in the relationship between teachers’ TL and SE. Therefore, Hypothesis 8 was proposed:

Hypothesis 8: Problem-based learning plays a mediating role in the relationship between TL and SE.

Problem-based learning is a trial-and-error process, as well as a learning context. In PBL, students try new problem-solving methods and derive new ways of thinking in different problem-solving contexts. In this process, key learning beliefs can be developed (Cai, 2013) that allow students to confidently face problems and challenges. Conversely, even if the learning context is insufficient in terms of depth and meaning, students’ high self-efficacy may buffer the low impact on SE caused by the deficient learning context (Dunlap, 2005), leaving students with advantageous SE. In the SCCT model, SSE plays a lubricating role in the learning process (van Dinther et al., 2011), enhancing the effect of PBL on SE. Therefore, SSE may have a mediating effect in the relationship between PBL and SE. Thus, Hypothesis 9 was proposed:

Hypothesis 9: Self-efficacy plays a mediating role in the relationship between PBL and SE.

**Figure 1:**
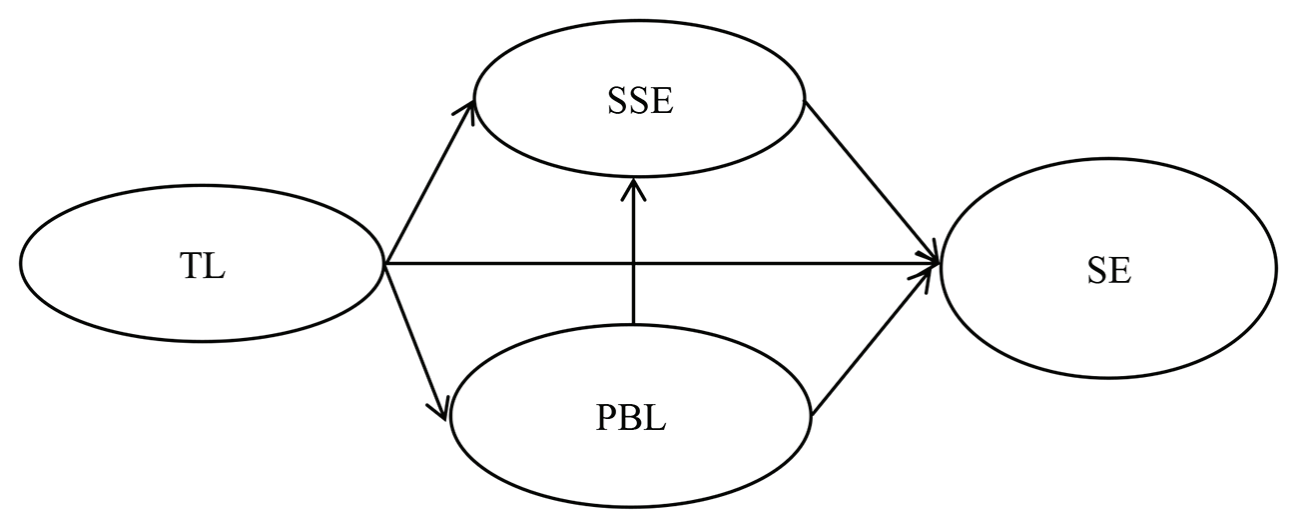
Research framework.

The research framework is shown in Figure 1.

## Methodology

### Participants and Sampling

This study involved conducting a questionnaire survey of university students in Taiwan. Due to the large number of HEIs in Taiwan, it is difficult to perform tests on all of the HEIs in the country; therefore, purposive sampling was employed. In addition, to accurately measure university students’ perceptions of the variables of the study and to enhance the study’s external validity, some principles for sampling were set. Firstly, junior and senior students who had adapted to university life were taken as respondents, as freshmen and sophomores may not be able to clearly express their employment intentions, making it impossible to measure the effect of each variable on SE. Secondly, considering that the sample needed to comprise of students with clear employment orientations, the question “Do you intend to pursue further study?” was included in order to exclude students who were less likely to seek work in the near future, enhancing the representativeness of the sample. Thirdly, as HEIs in Taiwan are generally classified into public and private universities, each type comprised half of the sample to enhance its representativeness.

Using telephone and email, the researchers initially contacted universities and teachers to enquire if they were willing to ask their students to complete the questionnaire. Before completing the questionnaires, students were asked if they understood their rights regarding the survey, in order to meet ethical requirements. A total of 1,000 questionnaires were distributed to 16 universities (seven public and nine private universities). A total of 637 valid questionnaires were returned, giving a response rate of 63.7%. The study considered university characteristics, including geographic location, school size, category, and attributes before the sampling to increase the generalizability of the study. Among the sample attributes, 383 were from public universities and 254 were from private universities; 321 were from northern universities, 185 were from central universities; and 131 were from southern universities. Furthermore, 49% (312) of the respondents were male and 51% (325) were female. In terms of family income, 17.3% of the respondents came from low-income families and 1.7% came from high-income families, and the rest were from well-off families (84.2%). Most of the students (74.5%) had not applied for a grant, and the study focused on respondents from the social sciences (55.7%; e.g., Business) and the natural sciences (44.3%; e.g., Technology, Engineering). This simplified the analysis process and kept the research focused. Due to the different types of HEIs and disciplines, a systematic error might have arisen, bringing the study’s external validity into question. Thus, several independent-samples *t*-tests were used to verify whether the groups of public universities vs. private universities and social sciences vs. natural sciences differed significantly in terms of the research dimensions. The results indicated that the groups did not significantly differ, so it was deemed appropriate to merge the samples from different universities and disciplines.

### Instrument

Student employability is a socio-psychological construct that includes subjective and objective aspects (De Vos et al., 2011). This study included the general ability for work (GAW; eight items), professional ability for work (PAW; four items), attitude at work (AW; three items), and career planning and confidence (CPC; three items) measures, as proposed by Pan and Lee (2011).

Teachers’ TL measurement items were revised based on the Multifactor Leadership Questionnaire (MLQ5X) prepared by Bass and Avolio (2000). The wording of this scale was modified from that of the study of Pounder (2008) to fit the classroom setting. Thus, it included II (four items), IS (four items), IC (four items), and IM (four items).

Student self-efficacy is an individual’s perception that they will achieve a goal before starting the necessary tasks. Student self-efficacy has a considerable influence on the choice of tasks, level of task performance, effort made to finish tasks, and persistence regarding task performance. The scale developed by Rigotti et al. (2008) was revised to integrate six items of higher reliability and validity.

For PBL, the scales developed by Chang et al. (2012) were adopted: KS (three items) and PS (three items). All items were measured on a five-point Likert scale (1 = totally disagree; 5 = totally agree) and are shown in Table 1.

**Table 1:** Instruments description.

Construct	Variables	Items
Student employability	General ability for work	Expression and communication
		Time management
		Leadership
		Innovation
		Team work
		Native language
		Foreign language
		Stability and pressure resistance
	Professional ability for work	Professional knowledge and skill
		Computer literacy
		Application of theory to work
		Problem finding and solving
	Attitude at work	Learning desire
		Plasticity
		Understanding of professional ethics
	Career planning and confidence	Understanding and planning of individual career development
		Understanding of environment and development of industries
		Job search and self-promotion
Problem-based learning	Knowledge sharing	Organized and prepared for small group sessions.
		To share thoughts and opinions with peer actively.
		To share all sources for picture, text, and other information.
	Problem solving	Utilizes relevant resource materials effectively.
		Utilizes internet or evidence-based materials to get appropriate information.
		Applies knowledge to new situations to solve problems and to reach decisions.
Self-efficacy	Self-efficacy	I can remain calm when facing difficulties in my job because I can rely on my abilities
		When I am confronted with a problem in my learning tasks, I can usually find several solutions.
		Whatever comes my way in my learning tasks, I can usually handle it.
		My past experiences in my learning tasks have prepared me well for my occupational future.
		I meet the goals that I set for myself in my learning tasks.
		I feel prepared for most of the demands in my learning tasks.
Teachers’ transformation leadership	Idealized influence	Teacher makes me proud to being associated with him/her.
		Teacher has a “sense of mission” which he/she transmits to me.
		Teacher displays conviction in his/her ideas, beliefs, and values.
		Teacher specifies the importance of having a strong sense of purpose.
	Intellectual stimulation	Teacher asks me to re-examine critical assumptions to questions whether they are appropriate.
		Teacher seeks differing perspectives when I am solving problems.
		Teacher gets me to look at problems from many different angles.
		Teacher challenges me to think about problems in new ways.
	Individualized consideration	Teacher spends time teaching and coaching me.
		Teacher treats me as a partner.
		Teacher considers that I have different strengths and abilities from others.
		Teacher helps to develop my own strengths.
	Inspirational motivation	Teacher talks optimistically about future with me.
		Teacher talks enthusiastically about my needs.
		Teacher expresses confidence to achieve objectives.
		Teacher articulates a compelling vision of the future.

### Procedure

This is a cross-sectional study whose research framework and survey instrument have been approved by the Institutional Review Board of University of Taipei. The researchers contacted colleges and teachers who were willing to receive the questionnaire by telephone and email first. The survey packages were sent by post to students of 16 universities. Each survey package contained a covering letter explaining the survey purpose, a survey instrument, and a postage-paid envelope. Before filling out the questionnaires, the students were asked if they understood their rights when answering the survey to ensure research ethical aspects. The students voluntarily completed the questionnaires, after signing their informed consent. During the school year (September 2018 to January 2019), students completed the questionnaire.

### Data Analysis Strategy

This study tested the hypotheses of research framework and included paths *via* structural equation modeling (SEM). For constructs with a higher-order factor structure (TL, PBL, and SE), we reduced the number of parameters to be estimated following the partial aggregation method (Bagozzi and Edwards, 1998; Little et al., 2002). This procedure involves averaging the responses of subsets of items measuring a construct. Because TL, PBL, and SE were unidimensional constructs, we followed the procedure recommended by Little et al. (2002) to create two parcels of randomly selected items to serve as indicators for these variables. Structural validity analysis was performed using the IBM-AMOS statistical program, v. 23.0 (New York, NY, USA) for Windows; this program was also used to construct the structural prediction model, specifically, verification of the structural linear prediction hypothesis (path analysis; de la Fuente et al., 2020).

## Analysis and Results

### Assessing Measurement Model

All scales were found to be reliable, with Cronbach’s α values ranging from 0.83 to 0.96. In order to gauge the construct validity (both convergent and discriminant) of the scales, confirmatory factor analysis was employed using AMOS 23.0. Hair et al. (2006) recommended convergent validity criteria as follows: (1) standardized factor loading of higher than 0.5 (See Table 2), (2) average variance extracted (AVE) above 0.5, and (3) composite reliability (CR) above 0.7. The evaluation standard for discriminant validity is the square root of the AVE for one dimension greater than the correlation coefficient with any other dimension(s). As Table 2 indicates, all three criteria for convergent validity were met. In addition, although II-IS (*r* = 0.88) and GAW–PAW (*r* = 0.81) were higher than the square root of the AVE, both scenarios occurred in the same variable (TL and SE) and, as expected, the correlation coefficients were high. Most correlation coefficients were less than the square root of the AVE within one dimension, suggesting that each dimension in this study had good discriminant validity.

**Table 2:** Measurement model.

	1	2	3	4	5	6	7	8	9	10	11
1. II	0.878										
2. IS	0.881^**^	0.891									
3. IC	0.818^**^	0.823^**^	0.879								
4. IM	0.803^**^	0.791^**^	0.806^**^	0.879							
5. SSE	0.509^**^	0.520^**^	0.569^**^	0.534^**^	0.824						
6. KS	0.528^**^	0.510^**^	0.493^**^	0.515^**^	0.533^**^	0.881					
7. PS	0.469^**^	0.469^**^	0.545^**^	0.483^**^	0.650^**^	0.698^**^	0.881				
8. GAW	0.479^**^	0.474^**^	0.500^**^	0.507^**^	0.503^**^	0.529^**^	0.546^**^	0.780			
9. PAW	0.466^**^	0.460^**^	0.504^**^	0.497^**^	0.502^**^	0.497^**^	0.522^**^	0.814^**^	0.856		
10. WA	0.532^**^	0.515^**^	0.553^**^	0.543^**^	0.561^**^	0.528^**^	0.572^**^	0.757^**^	0.747^**^	0.857	
11. CPC	0.532^**^	0.510^**^	0.526^**^	0.537^**^	0.558^**^	0.500^**^	0.532^**^	0.656^**^	0.640^**^	0.744^**^	0.888
Means	3.608	3.616	3.687	3.612	3.754	3.536	3.761	3.536	3.642	3.605	3.557
SD	0.728	0.730	0.720	0.724	0.624	0.741	0.701	0.640	0.701	0.704	0.726
Crobach’s *α*	0.901	0.913	0.901	0.901	0.905	0.856	0.857	0.906	0.878	0.802	0.865
AVE	0.771	0.793	0.772	0.772	0.679	0.777	0.777	0.608	0.733	0.718	0.788
CR	0.931	0.939	0.931	0.931	0.927	0.912	0.913	0.925	0.916	0.884	0.918

** If *p* < 0.01.

The diagonal value is the square root value of average variance extracted (AVE).

### Examination of the Structural Model

In this study, the measurement patterns of the abovementioned potential variables were established according to the research framework, and the model-matching degree of the SEM verification theory was adopted. For mode-matching tests, Bagozzi and Yi (1988) stated that the size of the sample should be considered, suggesting that when the mode fit is measured by the ratio of *χ*^2^ to the degrees of freedom (*df*), it generally does not exceed 3 (Hair et al., 2010). In this study, 619 valid questionnaires were analyzed. The ratio of *χ*^2^ to its df (2.47) was less than 3; PNFI (0.7) was greater than 0.5; goodness of fit index (GFI) was 0.97; adjusted goodness of fit index (AGFI) was 0.94; normed fit index (NFI) was 0.98; comparative fit index (CFI) was 0.99; and incremental fit index (IFI) was 0.99; thus, all were greater than 0.9. In addition, the root mean square error of approximation (RMSEA) was 0.05 and, hence, less than 0.06. Thus, the moderation of this research model is acceptable.

### Verification of Structural Model

The correlations among the variables were identified using SEM. There are many items in the consideration of certain facet scales. If a single question is used as the observation index for analysis, the model will become complicated, and the number of samples required for analysis will also be relatively small. Inflated, coupled with the fact that some topics may deviate significantly from normal distribution, resulting in low fitness, the study used item parceling for the SEM (Little et al., 2002). That is, the research topic items with the highest loadings were combined with those with the lowest loadings, those with the second highest were combined with those with the second lowest, and so on.

**Figure 2:**
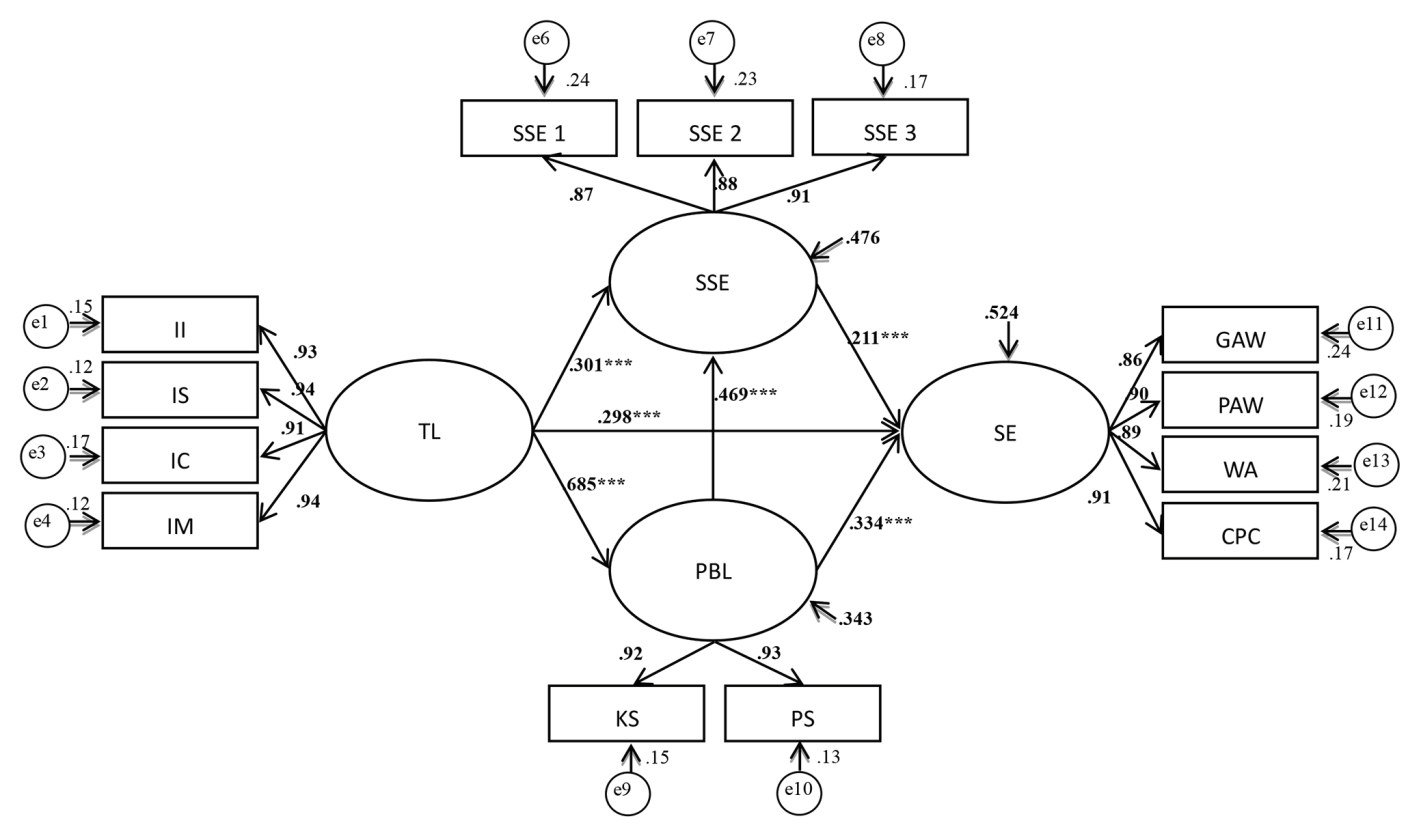
Structural model.

The findings are shown in Figure 2. The path coefficient of teachers’ TL to SSE was 0.230 (*t* = 5.106, *p* < 0.001), so Hypothesis 1 was supported. The greater the depth and breadth of teachers’ TL, the more confident the students are in their learning. The path coefficient of teachers’ TL to SE was 0.280 (*t* = 6.406, *p* < 0.001). This supports Hypothesis 2, indicating that as the content of teachers’ TL matures, students’ ability to absorb and apply knowledge to enhance their employment skills increases. Additionally, the path coefficient of teachers’ TL to PBL was 0.632 (*t* = 15.33, *p* < 0.001); thus, the higher the degree of TL, which represents students’ perceptions of the teacher, the higher the students’ involvement in PBL. Therefore, Hypothesis 3 was supported. The path coefficient of SSE to SE was 0.172 (*t* = 3.191, *p* < 0.001), so Hypothesis 4 was also supported. The results indicate that if students have greater self-efficacy toward achieving learning tasks and objectives, this will be conducive to improving their employability. The coefficients of PBL to SSE and SE were 0.590 (*t* = 11.33, *p* < 0.001) and 0.430 (*t* = 6.91, *p* < 0.001), respectively, so Hypothesis 6 and Hypothesis 7 were also supported.

The normalized effect values of the direct, indirect, and total effects of the facets were collated, as shown in Table 3, and hypothesis verification regarding the meditating effects was performed accordingly. The path coefficient of the indirect effect on SE through SSE and PBL was 0.375. Based on suggestions by Shrout and Bolger (2002), the ratio of indirect effect and total effect was used as the evaluation index of the indirect effect’s intensity; this showed that the intensity of the indirect effect was much greater than that of the direct effect (0.280). This indicates that the indirect effect plays an important role and also confirms that SSE and PBL have mediating effects in the relationship between TL and SE. Therefore, Hypothesis 5 and Hypothesis 8 were supported. Furthermore, the indirect effect of self-efficacy for PBL and SE was 0.101, indicating the important mediating role that SSE plays and supporting Hypothesis 9.

**Table 3:** Path coefficient of direct, indirect, and total effects.

Construct	Effects	SSE	PBL	SE
TL	Direct effect	0.301	0.685	0.298
	Indirect effect	0.274	—	0.316
	Total effect	0.575	0.685	0.615
SSE	Direct effect	—	—	0.211
	Indirect effect	—	—	—
	Total effect	—	—	0.172
PBL	Direct effect	0.469	—	0.334
	Indirect effect	—	—	0.099
	Total effect	0.590	—	0.433

## Discussion

In previous discussions of SE, many studies have focused on the antecedents of students’ individual behavior patterns and psychological cognition (Ahmed et al., 2015; Cacciolatti et al., 2017; Blázquez et al., 2018). However, this study aimed to verify whether teachers’ leadership style positively affects SE, assuming that teachers’ TL has a direct effect on PBL, SSE, and SE. The results support this assumption. Based on the employability concept in SCCT, the positive concept was interpreted through teachers’ TL. Through goal-setting and individual attention, students can achieve greater learning motivation and can adopt effective learning methods. According to the above arguments, TL can be regarded as an important resource to support student learning. When students receive knowledge and information transmitted by TL, they are willing to share this knowledge and solve problems through social interaction, thus promoting their SE.

Unlike research of Dacre Pool and Qualter (2013), the current study took junior and senior students as the subjects, rather than exploring the relationship between the self-efficacy and employability of serving staff. Therefore, the results overcome the lack of student samples in the SCCT satisfaction model and employability theory. As employability is a psychosocial facet, it can be explained through the social cognitive variables of self-efficacy, which conforms to research by Bandura (1995). Additionally, with the establishment of strong self-efficacy, individuals can enhance their ability to organize, manage, and execute tasks; in this way, this study has developed a socialized model of learning and of enhancing SE.

The results reveal the positive and direct impact of PBL on the development of SE. That is, the design of learning contexts is highly correlated with employability and stimulates positive behaviors and abilities. A reason for this may be that PBL makes students construct their own knowledge and abilities, so prior knowledge and experience can be meaningfully linked with newly acquired knowledge. Although PBL can significantly affect the acquisition of professional knowledge, no consistent outcome has been found for student achievement (White et al., 2004). This may be due to differences in the research samples, leading to the identification of the varied needs of different professional fields for PBL.

This study also explored the mediating mechanism between teachers’ TL and SE. The results show that teachers’ TL improves SSE and PBL, and this intensified SSE and PBL will contribute to SE. This conclusion is consistent with the findings of Lent et al. (2012) that SSE and PBL play important roles in the SCCT model and have an influence on the development of SE that cannot be ignored.

## Implications

This study explored teachers’ TL from the perspective of students. Student employability was used as a dependent variable, and PBL and SSE were used as mediators to test their effects on SE development. The researchers propose three implications as a reference for universities and teachers in guiding students to develop their employability. Firstly, universities are encouraged to provide information related to teachers’ TL and its application in combination with teachers’ professional development. Alternatively, they could analyze the differences in information and formulate professional development programs for teaching by attaining an understanding in advance of teachers’ self-evaluated leadership styles and students’ assessments of teaching. The results also show that teachers’ TL has significant positive relationships with SSE, PBL, and SE. In the literature, few studies have examined teachers’ TL as an important antecedent to the SCCT model, linking teachers’ leadership styles to students’ psychological status and learning performance (Pounder, 2008, 2014; Robinson et al., 2008; Shatzer et al., 2013). As teachers’ TL focuses on students’ perceptions of teachers’ care and guidance, as well as teaching goals, understanding students’ cognition of TL should be the focus of planning leadership practices in the future, in order to continuously provide references for teachers’ leadership styles and adjustments to teaching activities.

According to Bandura (1997), self-efficacy can be improved through subjective experiences, alternative experiences, verbal persuasion, and emotional states. All of these antecedent factors, except subjective experiences, can be obtained in the interactive process of TL and PBL. Thus, this study suggests that teachers should encourage students as another source of confidence, besides their families and peers. According to Lent et al. (1994), self-efficacy is the key structure of SCCT and is believed to have a direct impact on behavior. This implies that SSE can be seen as an important cognitive variable in the process of interpreting an individual’s formative behaviors, alongside interaction with the environment (Lent et al., 2014; Sheu et al., 2014).

It is necessary to implement strategies to increase students’ confidence in solving problems in order to reduce frustration from learning difficulties or lack of ability. This study suggests that universities and teachers should establish KS learning environments so that students can consider problems from different perspectives. Furthermore, teachers should encourage students to solve problems through experience-sharing and teamwork. This would help students to increase their engagement in various learning processes and gain the knowledge required to improve their employability, such as using mobile information technology to search for and discuss data.

## Research Limitations and Future Suggestions

This study contributes to the literature on SCCT, leadership theory, and SE. However, there were several limitations that represent directions for future research. Firstly, TL has previously received considerable attention in the business management field. However, less attention has been paid to the relationships among teachers’ TL, PBL, SSE, and SE within higher education. Although the research framework was constructed using SCCT, and the results provide important findings for learning theories, other theories could be used to explain how to enhance students’ learning ability and effectiveness, such as knowledge management theory, motivation theory, and demand hierarchy theory. Therefore, future studies are recommended using different theoretical models to consider the relevant knowledge source aspects that affect SE.

Secondly, this study only investigated Taiwanese students to verify the research framework. However, Taiwan shares many higher education similarities with different regions, such as mainland China, Malaysia, Hong Kong, and Thailand. This implies that cross-region comparisons may bring more insights to support the research results and form a comprehensive judgment. Future research could explore and compare other groups, in addition to expanding the sample size and improving the research representativeness, in order to provide additional insights relevant to higher education policy.

Thirdly, learning performance was used as an employability indicator, mainly because data on actual employability are not easy to obtain due to personal privacy reasons. In future, if the practical academic achievements of students could be considered while respecting research ethics, we may be able to better understand the relationship between the learning context and learning ability.

Fourthly, due to time and space constraints, only 12 universities were considered in this study, with 619 valid questionnaires and an undifferentiated study area. Scholars believe that gender is also an important factor affecting SE; thus, in addition to expanding the sample size to improve the research representativeness, multi-group discussions or comparisons should be conducted to propose pluralistic and in-depth policies for higher education.

## Data Availability Statement

The raw data supporting the conclusions of this article will be made available by the authors, without undue reservation.

## Ethics Statement

The studies involving human participants were reviewed and approved by Institutional Review Board, University of Taipei. The patients/participants provided their written informed consent to participate in this study.

## Author Contributions

This study is a joint work of the all authors. XL and MP contributed to the ideas of educational research, collection of data, and empirical analysis. MP, MA, and W-LC contributed to the data analysis, design of research methods, and tables. MP and BL participated in developing a research design, writing, and interpreting the analysis. All authors contributed to the literature review and conclusions.

### Conflict of Interest

The authors declare that the research was conducted in the absence of any commercial or financial relationships that could be construed as a potential conflict of interest.
